# Nanocomposites: Nanoscience and Nanotechnology (Nanoscale Phenomena) in Advanced Composites

**DOI:** 10.3390/nano12010081

**Published:** 2021-12-29

**Authors:** Muralidharan Paramsothy

**Affiliations:** NanoWorld Innovations (NWI), 1 Jalan Mawar, Singapore 368931, Singapore; mpsothy@yahoo.co.uk

Nanocomposites can be viewed as abundant interface nanoscale systems having the ability to manipulate length scales that are fundamentally important to many physical properties. This can sometimes impart multiple functions, which has led to a rapidly expanding scope and arena of application for these materials. It is known that the function of nanoparticles in a matrix is dependent on nanoparticle–matrix interactions, amongst other factors. This is with regard to any material property of the nanocomposites, be it related to crystallographic and/or electronic structure. In line with this theme, eight representative articles [1,2,3,4,5,6,7,8] have been published in this Special Issue:

Research by Mi et al. [1] was the first to simulate and analyse the dielectrophoretic motion of boron nitride nanosheets (BNNS) particles in ultrapure water under a nanosecond pulse voltage and propose a simulation theory of particle dielectrophoretic motion under a pulse voltage. The results showed that when the pulse voltage was applied, the relaxation polarisation time of the particles and the fluid needed to be considered and compared with the nanosecond pulse width. This was the fundamental difference from a direct voltage. The theory of particle dielectrophoretic motion under a pulse voltage proposed can provide a theoretical basis for experiments on pulsed electric-field-induced particle orientation and arrangement. The simulation model can be used as an effective tool for the simulation of any dielectric whose relaxation polarisation time is much shorter than the pulse voltage width.

Karunakaran et al. [2] reported the synthesis of Fe–Co–Ni ternary nanocomposites obtained by co-precipitation and followed by hydrogen reduction, and the effect of Fe content on the consequent magnetic properties (as a first report). Initial hydroxides of Fe-Co–Ni combinations were prepared by the co-precipitation method from nitrate precursors and precipitated using alkali. The reduction process was carried out by hydrogen in the temperature range of 300–500 °C under isothermal conditions. The nanocomposites had metallic and intermetallic phases with different lattice parameter values due to an increase in Fe content. It was shown that the values of the magnetic parameters of nanocomposites could be controlled in the ranges of M_S_ = 7.6–192.5 Am^2^/kg, M_r_ = 0.4–39.7 Am^2^/kg, M_r_/M_s_ = 0.02–0.32, and H_cM_ = 4.72–60.68 kA/m, by regulating the Fe content and reduction temperature of Fe–Co–Ni nanocomposites. The reduction in the surface spin fraction of the nanocomposites under growth induced an increase in saturation magnetisation. The Fe–Co–Ni ternary nanocomposites are superior magnetic materials for various magnetically coupled device applications.

Importantly, in the context of green research, Du et al. [3] successfully fabricated Co_3_O_4_–modified TiO_2_ nanorod arrays using green photochemical deposition methods without adding any toxic reagents. The Co_3_O_4_–TiO_2_ nanorod arrays fabricated in acid solution had the highest photo-electrochemical activity. The mechanism of Co_3_O_4_–TiO_2_ nanorod formation with pH was investigated. The morphology of Co_3_O_4_ was controlled by adjusting the concentration of Co^2+^ ions. Low concentrations of Co^2+^ ions formed isolated islands of Co_3_O_4_ supported on TiO_2_ nanorods. These Co_3_O_4_ islands acted as active catalytic sites to mineralise organic wastes during photoelectrochemical degradation. The Co_3_O_4_–TiO_2_ nanorod arrays exhibited higher photoelectrochemical activity to degrade organic waste, compared with pure TiO_2_ nanorod arrays, which demonstrates great potential in wastewater treatment.

In a work by Polizos et al. [4], styrene-based elastomer composites with graphene oxide (GO) and silica nanofiber (SnF) fillers were synthesised. The surface of the GO fillers was modified using two functionalities: cysteamine functionality to introduce sulphur groups (SH) onto reduced GO (rGO) and form covalent bonds with the styrene–butadiene rubber (SBR) matrix and dodecylamine (DA) functionality to introduce hydrophobic groups onto GO and allow the liquid mixing of GO–DA and SBR in toluene. Oxygen reduction occurred simultaneously during the cysteamine functionalisation. The oxygen content was reduced from approximately 26 at% in the pristine GO to 16–18 at% in the cysteamine functionalised GO. The intercalated cysteamine increased the basal plane d-spacing of the GO by 4.5 Å. The SBR composites filled with rGO–SH, and GO–DA were prepared using mechanical and liquid mixing techniques, respectively. The storage modulus of the SBR/GO-DA composite increased by approximately 60% for temperatures lower than −40 °C and nearly 200% for temperatures higher than 20 °C, compared with the SBR/rGO–SH, due to better filler dispersion and interfacial adhesion between GO and SBR. The latter was also evident by the shift toward higher temperatures of the relaxation mechanism associated with the glass transition of the interfacial SBR layer. Synergistic improvements due to the complementary properties of the GO–DA and SnF fillers were achieved in the hybrid SBR/SnF/GO-DA composite. The storage modulus further increased by approximately 97% and 80% for temperatures lower than −40 °C and higher than 20 °C, respectively, compared with the SBR/GO–DA composite. SBS composites were prepared at 1, 3, 5, and 10 wt% GO. The maximum stress and modulus values increased almost linearly for the weight loadings up to 5 wt%. The SBS 5 wt% GO showed a 3.7-fold increase in the maximum stress and a 6-fold increase in the modulus, compared with the unfilled SBS. The increase in the thermal conductivity of the SBR and SBS composites with comparable GO weight percent was similar. The highest value was 0.38 W/mK for the SBS filled with 5 wt% GO, which corresponded to an 86% increase with respect to the unfilled SBS.

Concerning dental research, Babina et al. [5] investigated the effect of final surface treatment and dental composite type on the roughness of the composite surface, composite–enamel interface, and composite–cementum interface, as well as on the polishing time. Class V cavities prepared in extracted teeth (n = 126) were restored using one of the three nanohybrid composites with different filler sizes. The specimens were randomly assigned to three different finishing and polishing sequences, roughness (Ra) of the investigated surfaces was measured using profilometry, and the time taken to achieve visible gloss was documented. The data were analysed using ANOVA with Tukey’s post hoc test (*p* < 0.05). There was no significant influence of the composite type on the restoration surface roughness (*p* = 0.088), while the polishing method had a significant impact (*p* < 0.001). The Ra of the composites ranged between 0.08 µm and 0.29 µm, with the lowest values (0.09 µm ± 0.05 µm) found in the aluminium oxide disc group (*p* < 0.001). The time to achieve a visible composite gloss was influenced by the polishing method, composite type, and interactions between these factors (*p* < 0.001). The interface roughness was significantly greater than that of the composite surface (*p* < 0.001), dependant on the composite type and polishing system employed.

Gao et al. [6] reported the development of a new method for flux-free diffusion joining of aluminium matrix composites reinforced with SiC particles (SiCp/Al MMCs) in an atmospheric environment. Liquid gallium and nanocopper particles were used as filler metal under joining temperatures ranging from 400 °C to 480 °C, with a holding time of 2 h and pressure of 3 MPa. The results showed that 65 vol% SiCp/6063 Al MMCs were successfully joined together. X-ray diffraction (XRD) analysis confirmed the presence of Ga_2_O_3_ at the fracture. Meanwhile, neither copper oxide nor aluminium oxide was detected. The formation of Ga_2_O_3_ protected nanocopper particles and SiCp/6063 Al MMCs from oxidation. The weld seam width was reduced from 40 µm to 14 µm gradually, with increasing temperature of 400 °C to 480 °C. The maximum shear strength of 41.2 MPa was achieved with a bonding temperature of 450 °C. The increase in strength was due to the adequate interdiffusion and solid solution formation between the elements, as well as the change in quantity and morphology of intermetallic compounds such as Al_2_Cu and Cu_3_Ga in the weld seam. When the diffusion joining temperature reached 440 °C or above, the leak rate of the specimen remained under 10^−10^ Pa·m^3^/s.

Critically for biomolecular design and drug delivery application, Feng et al. [7] encapsulated typically water-insoluble curcumin in an amphiphilic biopolymer known as octenyl succinic anhydride–short glucan chains (OSA–SGC), using self-assembly, to form water-soluble curcumin–OSA–SGC nanoparticles. Curcumin displays anti-cancer, anti-inflammatory, and anti-obesity properties, but its water insolubility limits the wholesome utility. The nanoparticles were prepared with the degree of substitution (DS) of 0.112, 0.286, and 0.342. The nanoparticles prepared were in the diameter range of 150–200 nm and displayed high encapsulation efficiency and loading capacity of curcumin. DS had little effect on the total amount of curcumin encapsulated. High encapsulation efficiency of 64.62% was observed for the DS value of 0.342. The lower release rate of the curcumin, from the curcumin–OSA–SGC nanoparticles, in simulated intestinal conditions, opened up novel opportunities to deliver health-promoting and disease-preventing compounds in the intestinal tract. These nanoparticles have the potential to deliver several water-insoluble functional ingredients.

Last but not least, Nouari et al. [8] reviewed spark plasma sintered (SPS) alumina hybrid nanocomposites, including potential applications and future directions. Ceramics have many advantages, compared with metals in specific applications, and can be more widely applied if the relatively inferior properties (fracture toughness, tensile strength, electrical and thermal conductivities) are improved. Reinforcing ceramics by two nano-phases that have different morphologies and/or properties, called the hybrid microstructure design, has been implemented to develop hybrid ceramic nanocomposites, with tailored nanostructures, improved mechanical properties, and enhanced functional properties. The use of the novel SPS process allowed for the sintering of hybrid ceramic nanocomposite materials to maintain high relative density while also preserving the small grain size of the matrix. Consequently, hybrid nanocomposite materials having better mechanical and functional properties than those of either conventional composites or nanocomposites were produced. The development of hybrid ceramic nanocomposites is in its early stage and is expected to continue attracting the interest of the scientific community. The progress made in the development of alumina hybrid nanocomposites (using SPS) and material properties were reviewed. Current challenges and potential applications were highlighted. Future prospects for developing higher performance alumina hybrid nanocomposites were also addressed.

## Data Availability

Not applicable.
